# HIV-1 genetic diversity and pretreatment drug resistance survey prior to dolutegravir introduction in Senegal

**DOI:** 10.1016/j.ijregi.2025.100741

**Published:** 2025-09-05

**Authors:** Mengue Fall, Nafissatou Leye, Nicole Vidal, Fatou Niasse, Edmond Tchiakpe, Bambo Diakhaby, Mame Salane Thiam, Abou Abdallah Malick Diouara, Fabien Roch Niama, Safiatou Thiam, Coumba Toure-Kane, Halimatou Diop-Ndiaye

**Affiliations:** 1 University Cheikh Anta Diop of Dakar, Laboratory of Bacteriology-Virology CHU A. Le Dantec, Dakar, Senegal; 2 Institute of Health Research, Epidemiological Surveillance, and Training, Dakar, Senegal; 3 IRD_UMI 233 TransVIHMI, Délégation Régionale Occitanie 911 avenue Agropolis, Montpellier, France; 4 Conseil national de Lutte contre le SIDA, Dakar, Senegal; 5 University Abomey Calavi and National Reference Laboratory of Health Program Fighting Against AIDS in Benin (LNR/PSLS), Health Ministry of Benin, Cotonou, Benin; 6 Laboratoire National de Santé Publique, Unité de Biologie Moléculaire, Faculté des Sciences et Techniques, Université Marien Ngouabi, Brazzaville, Republic

**Keywords:** Pretreatment drug resistance mutations, Dried blood spots, Senegal

## Abstract

**Objectives:**

This study aimed to document the prevalence of pretreatment drug resistance in antiretroviral therapy-naïve patients in Senegal, describe mutation profiles, and evaluate their potential impact on future therapy.

**Methods:**

A pretreatment drug resistance survey was carried out in 35 sites throughout the country between 2017 and 2018 using dried blood spots and whole blood samples. Amplification and sequencing were performed on the partial reverse transcriptase (RT) gene using the Agence Nationale de Recherches sur le Sida et les hépatitis virales (ANRS: French National Agency for Research on acquired immunodeficiency syndrome [AIDS] and Viral Hepatitis)/AC11 method at the HIV National Reference Laboratory. Surveillance drug resistance mutations (SDRM) were analyzed using the Calibrated Population Resistance v8.1, and the proportion of SDRM was evaluated after grouping sites by geographical areas.

**Results:**

A total of 237 samples from different patients were analyzed, and 131 (55.3%) were successfully amplified and sequenced. Among these, 14 (10.7%) harbored SDRM with significant variability across regions. For nucleoside reverse transcriptase inhibitors (NRTI), the SDRM rate was highest in Dakar (9.1%), followed by the Southwest (5.3%) and the Southeast (4.2%). For non-NRTIs (NNRTIs), the highest rate was observed in the Southeast (20.8%), followed by Dakar (13.6%), the Midwest (8.3%), and the Southwest (3.6%). Among NRTIs, the most frequent mutation was M184V (3/6), conferring high-level resistance to lamivudine and emtricitabine, and K103N (11/12) among NNRTIs, conferring resistance to efavirenz and nevirapine. Notably, four sequences harbored SDRMs for both NRTI and NNRTI, reflecting overlapping resistance in some patients. The higher prevalence of NNRTI resistance is consistent with the lower genetic barrier of these drugs compared to NRTIs, which may facilitate the emergence of pretreatment drug resistance.

**Conclusion:**

Through a quasi-national survey, an intermediate level of NNRTI resistance was observed, with regional disparities in Senegal. These findings highlight the limitations of NNRTI-based first-line regimens in Senegal and support the transition to other antiretroviral therapy regimens, such as dolutegravir-based therapy in newly infected individuals as well as in treated patients.

## Highlights

• Intermediate prevalence of non-nucleoside reverse transcriptase inhibitor drug resistance in HIV-1 patients prior to TLD introduction in Senegal.
• Regional disparities in surveillance drug resistance mutation rates in Senegal highlighted the importance of tailored approaches.
• There is a need to support the transition to a non-nucleoside reverse transcriptase inhibitor-free-based regimen, such as dolutegravir-based therapy.

## Introduction

Antiretroviral therapy (ART) has revolutionized the outcome for people living with HIV (PLHIV), especially in sub-Saharan Africa, with a 69% reduction in HIV-related deaths from 2 million in 2004 to less than 700,000 in 2020 (https://www.hiv.gov/hiv-basics/overview/data-and-trends/global-statistics/, accessed December 2023). However, a major limitation of ART is the risk of the emergence of drug resistance mutations (DRMs), which can render antiretroviral drugs ineffective and lead to therapeutic failure [[1], [2]]. These resistance mutations can also be transmitted to ART-naive individuals, resulting in pretreatment drug resistance (PDR) that may compromise the efficacy of first-line ART [3].

The emergence of DRMs is influenced by the antiretroviral (ARV) drugs used. Until 2018, World Health Organization (WHO) recommendations for first-line ART were based on a combination of two nucleoside reverse transcriptase inhibitors (NRTIs) and one non-NRTI (NNRTI) [3]. These regimens, widely used in resource-limited countries, have shown limitations due to the occurrence of resistance mutations, leading to treatment failure. For instance, in Senegal, several studies have reported increasing rates of virological failure and resistance after first-line ART in both the capital and decentralized health settings [[4], [5], [6], [7], [8]]. In the West African sub-region, similar findings have been reported, with virological failure and ARV resistance ranging from 18.1–25% in some areas [[7], [8], [9]]. Such DRMs clearly impact future therapeutic options, highlighting the need for ongoing surveillance [10,11].

Surveillance of the level of HIV drug resistance (HIV DR) in a given population is one of the key tools developed by WHO to monitor the effectiveness of ART. In adult patients, this surveillance could be for acquired drug resistance (ADR) in populations receiving ART or Surveillance of PDR in populations initiating ART with or without prior antiretroviral drug exposure (https://www.who.int/publications/i/item/978-92-4-151284-8, accessed December 2023). ADR occurs when HIV mutations emerge due to ongoing viral replication in people receiving ARV drugs, while PDR is detected in people who have never received ARV drugs and have no history of ARV drug exposure (https://www.who.int/publications/i/item/978-92-4-151284-8, accessed December 2023).

HIV genetic diversity is high in West Africa, with CRF02_AG predominating at over 60% [6,[12], [13], [14], [15], [16], [17], [18], [19], [20], [21]]. Some studies have also reported the emergence of subtype C among men having sex with men [22] and in the general population [13]. Because NNRTIs have a low genetic barrier to resistance and ARVs are also used in children for post-exposure prophylaxis, monitoring circulating HIV strains is crucial [17].

In 2018, Senegal conducted its first nation-wide survey following WHO guidance to assess levels of PDR and ADR, and to identify factors associated with the emergence of drug resistance.

This study aimed to document the prevalence of PDR in ART-naive patients in Senegal, describe the DRMs observed, evaluate their potential impact on future therapeutic options, and gain insights into the genetic diversity of HIV strains circulating in the country.

## Material and methodology

### Site selection and study population

PDR surveillance was carried out between 2017 and 2018 according to WHO recommendations (https://apps.who.int/iris/handle/10665/204471, accessed December 2023). In order to have a nationally representative sample, recruitment sites were randomly selected among those reporting more than 10 new PLHIV treated per year. Out of 129 healthcare centers in the country, 112 sites met this eligibility criterion. The final selection was made using WHO randomization software, which resulted in the selection of 35 sites throughout the country for surveillance of PDR, with a target of 331 patients to be included.

The inclusion criteria for patients in the sites were to be HIV-1-positive men or women, aged at least 18, ART-naive (defined as never having received any antiretroviral therapy, including prevention of mother-to-child transmission [PMTCT] regimens), and newly diagnosed based on the patient and the healthcare provider declarations.

In accordance with WHO recommendations, survey weights were applied to adjust prevalence estimates. These weights included a site sampling weight, calculated based on the size of the selected sites. These survey weights ensure that national-level estimates are representative and accurate.

For the study, the information leaflet and the consent form were submitted to the Senegalese ethics committee for approval, and the administrative authorization was obtained from the Senegalese Ministry of Health and Social Action. An information letter explaining the aim and expected results of this work was given to patients in order to obtain their consent. Only patients who gave free and informed consent were included in the study.

### Sample collection

One type of sample (dried blood spots [DBS] or venous blood) was used for each consenting patient. In Dakar (capital city), sample collection involved taking venous blood samples on ethylenediaminetetraacetic acid (EDTA) tubes from consenting patients. These samples were transported with an ice pack to the HIV National Reference Laboratory (NRL) of the Bacteriology-Virology laboratory at University Hospital Center (UHC) Aristide Le Dantec, where they were centrifuged at 2500 rpm to recover the plasma, which was stored at –80°C until testing.

In other sites outside Dakar, venous blood samples on EDTA tubes were used to prepare 2 DBS cards by saturating dedicated circles of Whatman 903 paper with whole blood. These blood spots were dried overnight or for at least 3 hours at room temperature and preserved with desiccants at 20–37°C on site before being transferred within 15 days to the reference laboratory in Dakar. This transfer to the NRL was performed within a maximum of 10 days, when they were stored at –80°C until analysis.

### Genomic amplification and sequencing

From 200 µl of plasma or two spots of DBS, HIV-1 viral ribonucleic acid (RNA) was extracted on the Nuclisens easyMAG® platform (Biomerieux, France) according to the manufacturer's recommendations. The resulting RNA was used to synthesize complementary deoxyribonucleic acid (cDNA), which was amplified using the ANRS AC11 genotyping technique targeting the first 240 amino acids of RT. Primer pairs used for polymerase chain reaction were MJ3/MJ4 and A35/N35. Purified and quantified polymerase chain reaction products were sequenced by using Big Dye Terminator v3.1 (Life Technologies, Courtaboeuf, France), and electrophoresis was performed on the ABI 3100 Avant sequencer (Applied Biosystems, France) as previously described [6].

### Sequence editing, quality control, and resistance mutation analysis

The sequences were edited on DNAStar's Seqman 5.08 software (Lasergene, Konstanz, Germany) and/or Recall (https://recall.bccfe.ca/) to eliminate detection errors. Sequence quality control was performed according to WHO procedures for quality assurance of resistance genotyping tests (https://apps.who.int/iris/bitstream/handle/10665/259731/9789241512879-eng.pdf, accessed October 2023). Briefly, sequences were first used for quality control to eliminate sequences linked together with a very small genetic distance and hypermutated sequences (https://sequenceqc.bccfe.ca/who_qc, accessed October 2023). Sequence length and the presence of stop codons were then checked on the Stanford HIV database (https://hivdb.stanford.edu/hivdb/by-sequences, accessed October 2023).

The analysis of resistance mutations was carried out using the Calibrated Population Resistance v8.1 program (https://hivdb.stanford.edu/cpr/, accessed December 2023) in search of specific mutations monitored in pretreatment. The proportion of DRM was evaluated globally, and after grouping sites by geographical areas (Dakar, North, Midwest, South-West, and South-East), as shown in Table 1.

**Table 1:** Number of samples received by site and zone.

Zone	Selected sites	Type of sample	Specimen expected N	Specimen confirmed n (%)	Average delivery time to Laboratory of Bacteriology and Virology (LBV) (days)	Successfully genotyped n (%)
Midwest	CS DAROU KHOUDOSS	DBS	5	5 (100%)	68	2 (40%)
	CS MBOUR	DBS	7	6 (85.7%)	1	4 (66.6%)
	CS NIORO	DBS	14	10 (71.4%)	20	6 (60%)
	CS SOKONE	DBS	8	3 (37.5%)	2	2 (66.6%)
	CS PASSY^a^	DBS	3	0	NA	NA
	CS THIES	DBS	6	5 (83.3%)	4	0 (0%)
	EPS KAFFRINE	DBS	11	11 (100%)	12	1 (10%)
	EPS MAT FAWZEINI	DBS	2	3 (100%)	17	3 (100%)
	HOP ST JEAN DE DIEU	DBS	3	3 (100%)	3	1 (33.3%)
	PTA KAOLOACK	DBS	7	7 (100%)	15	5 (71.4%)
Dakar	CPS	DBS	8	7 (87.5%)	56	1 (14.3%)
	CS GASPAR CAMARA^b^	Blood	5	5 (100%)	6	3 (60%)
	CS KEUR MASSAR	DBS	4	4 (100%)	5	2(50%)
	CS MBAO	DBS	8	8 (100%)	5	4 (50%)
	HALD^a^	BLOOD	4	0	NA	NA
	CTA^b^	BLOOD	13	12 (92.3%)	1	7 (59%)
	EPS ROI BAUDOUIN	DBS	8	3 (45.8%)	1	0 (0%)
	EPS RUFISQUE	DBS	6	6 (100%)	7	4 (66.6%)
	PMI MEDINA	DBS	2	1 (50%)	9	1 (100%)
North	CHR SAINT LOUIS	DBS	4	4 (100%)	2	3 (75%)
	EPS LINGUERE^a^	DBS	8	0	NA	NA
	CS SAINT LOUIS^a^	DBS	3	0	NA	NA
	CS MATAM	DBS	4	3 (75%)	5	2 (66.6%)
South-east	CHR TAMBACOUNDA	DBS	5	12 (100%)	8	8 (51.1%)
	CS TAMBACOUNDA	DBS	14	2 (14.3%)	5	
	CS MAKA COULIBANTAN	DBS	3	3 (100%)	13	3 (100%)
	CS VELINGARA	DBS	17	17 (100%)	9	13 (76.5%)
South west	CS BOUNKILING	DBS	25	25 (100%)	10	15 (60%)
	CS DIOULOULOU	DBS	10	4 (40%)	10	4 (100%)
	CS SEDHIOU	DBS	11	10 (90.9%)	13	6 (60%)
	PTA ZIGUINCHOR	DBS	16	15 (93.8%)	36	13 (86.7%)
	UTA KOLDA	DBS	31	29 (93.5%)	9	18 (41.9%)
	CHR KOLDA	DBS	14	14 (100%)	54	
	TOTAL		331	237 (71.6%)		131 (55.3%)

a Sites that did not deliver samples

b Sites that supplied whole blood.

### Phylogenetic analysis

The newly determined sequences were combined with known representatives of the different subtypes, sub-subtypes, and circulating recombinant forms (CRFs) circulating in Africa, which were uploaded from the Los Alamos HIV Sequence database. A multiple sequence alignment (MSA) was performed using MAFFT version 7 (https://mafft.cbrc.jp/alignment/server/), then manually checked and end-trimmed under AliView. Alignment curation was performed with G-Blocks (http://molevol.cmima.csic.es/castresana/Gblocks_server.html).

From the new MSA, each sample sequence was submitted to a similarity plot analysis with Simplot v3.5.1. In the case of recombinant profiles, individual bootscan analyses were refined with a restricted group of reference sequences. Each recombinant sequence was cut into segments, and each segment was used to construct a maximum likelihood (ML) phylogenetic tree to confirm the subtype/CRF of the segment.

ML trees were inferred under the GTR+4G+I (General Time Reversible plus among-site rate heterogeneity and invariant sites) nucleotide substitution model as implemented in PhyML 3.0. ML trees are used to infer the evolutionary relationships between sequences. The best of subtree pruning and regrafting and nearest neighbor interchange heuristic options was selected, and approximate likelihood ratios were used as branch supports.

The final MSA included all HIV-1/M subtypes, sub-subtypes, and CRFs represented in this study. The final ML tree was inferred under GTR + F + R5 as the best-fit model according to the Bayesian information criterion (BIC) and 1,000 bootstrap resamplings using IQ-Tree v1.6.12. Consensus trees were edited with FigTree v1.4.4. Transmission clusters were defined based on short branch lengths (≤0.015) and high support values (>98%) as previously described [22].

### Statistical analysis

All statistical analyses were performed using descriptive statistics, including frequencies, means, and proportions, calculated with SPSS v16.0 and Epi Info v3.5.4. The proportion of patients with HIV drug resistance (HIVDR) mutations was calculated separately for each ARV drug class (NRTI, NNRTI) and by geographical area. These results were not extrapolated to the entire country due to potential biases.

The RT-generated sequences were deposited in the National Institutes of Health GenBank database under accession numbers PP280627 to PP280757.

## Results

### Characteristics of patients and specimens

**Figure 1:**
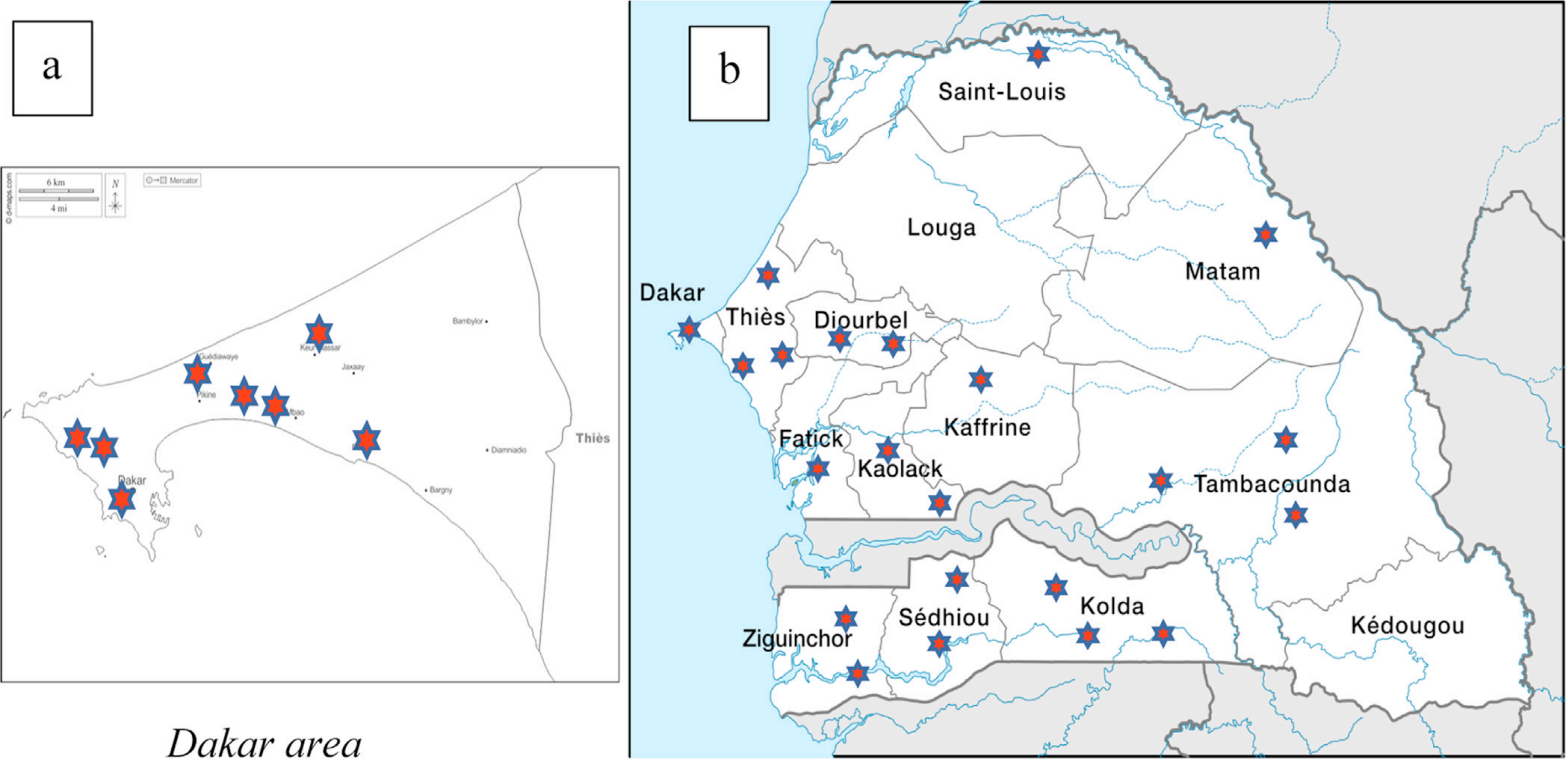
Cartography of selected sites in (a) Dakar and (b) the rest of the country.

A total of 251 of 331 ART-naive patients were recruited, giving a participation rate of 75.8%. After quality control, 237 samples were considered acceptable, giving a conformity rate of 94.4%. These patients were recruited from 32 sites across the country, including 8 sites from Dakar, the capital city (Figure 1). The other 14 samples were not included due to their quality (blood hemolysis) and the delay of the specimen transfer (after 15 days from sample collection).

The mean age of patients was 39 years (range: 18-75 years; SD = 14.25), and the sex ratio (M/F) was 0.61 (90 males and 147 females).

Most of the samples received were DBS (n = 121) originating primarily from the Southern part of the country with 97 (40.9%) and 34 (22.8%) samples from the Southeast and Southwest areas, respectively. Only 17 samples were received as whole blood. The average delivery time to the NRL was 14 days (1-68 days). The characteristics of patients are presented in Table 1.

### Genotyping results

Of the 237 samples, 131 (55.3%) were successfully amplified and genotyped on the RT gene, including 121 (55%) out of 221 DBS and 10 (76.9%) out of 17 plasma samples. The genotyping success rate varied by region, from 45.3% in the Midwest to 71.4% in the North (Table 2). Among the samples that could not be amplified, two were identified as HIV-2 by serology.

**Table 2:** Distribution of sequences with a SDRM according to the zones.

Zone	Specimen analyzed N (%)	Successfully genotyped N (%)	Sequences with SDRM n (%)	Sequences with NRTI SDRM n (%)	Sequences with non-NRTI SDRM n (%)
Dakar	46 (19.4)	22 (47.8%)	4 (18.18%)	2 (9.1%)	3 (13.6%)
Midwest	53 (22.4)	24 (45.3%)	2 (8.3%)	-	2 (8.3%)
North	7 (2.9)	5 (71.4%)	-	-	-
Southeast	34 (14.3)	24 (70.6%)	3 (12.5%)	1 (4.2%)	5 (20.8%)
Southwest	97 (40.9)	56 (57.7%)	5 (8.9%)	3 (5.4%)	2 (3.6%)
Total	237 (100)	131 (55.3%)	14 (10.7%)	6 (4.6%)	12 (9.2%)

NRTI, nucleoside reverse transcriptase inhibitor; SDRM, surveillance drug resistance mutation.

**Caption**: The percentage of SDRM were calculated using the number of samples analyzed as the denominator; the percentage of SDRM over than 5% were highlighted in grey.

Out of the 131 sequences obtained, 14 harbored at least one surveillance drug resistance mutation (SDRM), giving an overall PDR rate of 10.7% (14/131). The highest SDRM rates were observed in Dakar (18.2%; 4/22) and the Southeast zone (12.5%; 3/24), followed by the Southwest (8.9%; 5/56). No SDRM were detected in the Northern zone (Table 2).

Regarding ARV drug classes, NRTI SDRM prevalence was over 5% in Dakar (9.1%) and the Southwest (5.4%). NNRTI SDRM prevalence was highest in the Southeast (20.8%; 5/24) followed by Dakar (13.6%; 3/22), and only the Southwest area showed a rate below 5% (Table 2). Globally, out of 131 sequences, six showed SDRM for NRTI while 12 harbored SDRM for NNRTI, giving a rate of transmitted resistance in NRTI and NNRTI of 4.6% and 9.2%, respectively (Table 2).

### DRM analysis

Among NRTI SDRM, M184V was observed in three out of six patients. Other thymidine analogue mutations (TAMs) included M41L (n = 2), D67N (n = 1), K219Q (n = 1), K65R (n = 1), and T215S (n = 1). For NNRTI, K103N was the most frequent mutation (11/12), followed by P225H (n = 3), L100I (n = 2), Y181C (n = 1), and G190A (n = 1). Four patients harbored viruses with both NRTI and NNRTI SDRM.

The distribution of sequences with resistance mutations by geographical area is summarized in Table 3.

**Table 3:** Distribution of patients with a sequence presented at least one resistance mutation according to recruitment site.

Patient ID	Age	Sex	NRTI	NNRTI	Site	Zone
20559_HALD_RT	50	F	None	G190A	DAROU KHOUDOSS	Midwest
20308_HALD_RT	18	F	None	K103N, P225H	THIES	Midwest
20553_HALD_RT	25	M	None	K103N	DAKAR/CTA	Dakar
20555_HALD_RT	31	M	None	K103NS	DAKAR/CTA	Dakar
20547_HALD_RT	26	F	M41L	None	DAKAR/CTA	Dakar
20542_HALD_RT^a^	64	F	D67N, K219Q	K103N, P225H	DAKAR/GS	Dakar
20441_HALD_RT	28	F	None	K103N	VELINGARA	South-east
20437_HALD_RT	43	M	None	K103N	VELINGARA	South-east
20325_HALD_RT	45	F	None	K103N	TAMBA	South-east
20326_HALD_RT^a^	71	M	M184V	K103N, P225H	TAMBA	South-east
20329_HALD_RT	49	F	None	K103N, Y181C	TAMBA	South-east
20427_HALD_RT^a^	23	F	M41L, K65R, M184I	L100I, K103N	SEDHIOU	South-west
20521_HALD_RT	35	F	T215S	None	KOLDA	South-west
20511_HALD_RT^a^	44	M	M184V	L100I, K103N	KOLDA	South-west

a NRTI, nucleoside reverse transcriptase inhibitor; SDRM, surveillance drug resistance mutation. **Caption**: Samples with SDRM for NRTI and NNRT are flagged and mutations were highlighted in grey.

### Phylogenetic analysis

**Figure 2:**
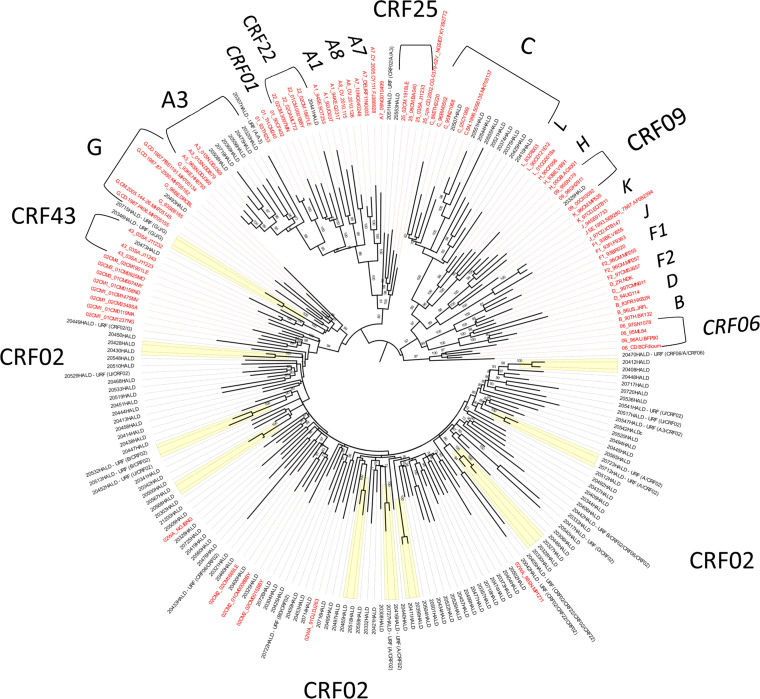
Distribution of strains according to RT subtypes.

Phylogenetic analysis of the RT gene showed a predominance of CRF02_AG (67.2%; 88/131), followed by subtype C (6.9%; 9/131), A3 (4.6%; 6/131), and single sequences for subtypes/CRFs: G, CRF09_cpx, CRF22_cpx, CRF25_cpx, and CRF43_02G. Additionally, 23 sequences (17.5%) were unique recombinants (URFs), composed of strains predominant or co-circulating in Senegal (Figure 2). Nineteen sequences (14.5%) clustered into nine transmission chains, mainly within the CRF02_AG (one triad and five dyads). Three dyads were also observed within URFs (two for URF (A/CRF02) and one for URF (G/J/G). One more dyad was noted for the URF (B/CRF02) as a borderline case since the average branch length was 0.016. Transmission clusters have been highlighted in the tree. Transmission clusters did not harbor any SDRM.

Maximum likelihood consensus tree derived from partial reverse transcriptase sequences (714 unambiguously aligned nucleotide sequences from 131 samples). Phylogenetic tree analysis was performed with 1000 bootstrap resamplings under the GTR + F + R5 as the best-fit model of nucleotide substitution according to BIC. The tree was edited with FigTree v1.4.4 with mid-point rooting. Reference sequence names are in red, the sequences from the study are in black. Transmission clusters are highlighted in yellow.

## Discussion

Drug resistance surveillance is mandatory for any HIV treatment program as recommended by WHO due to the risk of the emergence of DRM that will impact the treatment efficacy (https://www.afro.who.int/sites/default/files/2019-08/HIV_DrugRes_160819_FR.PDF, accessed December 2023). Routine surveillance of HIV drug resistance provides evidence that informs national HIV treatment guidelines and can be leveraged to optimize patient- and population-level treatment outcomes.

This study aimed to determine the prevalence of PDR in HIV naive patients before starting ART in Senegal and to analyze the implications of DRM for future therapeutic options.

Senegal was one of the first Sub-Saharan African countries to introduce highly active ART in 1998 through the Senegalese initiative of antiretroviral access, with a scale-up of ART starting in 2003 and free access for all PLHIV [23]. Numerous studies have monitored DRM in treated cohorts, both adults [[24], [25], [26]] and children [27,28]. However, few previous studies reported data from naïve populations [11,12], and this study is the first designed as a nation-wide HIV DR survey according to WHO recommendations to fill the knowledge gap.

Despite the follow, nearly one-fourth (24.2%) of expected samples were not received due to challenges in recruiting naive patients. Samples were ultimately collected from 32 sites out of the 35 initially planned. In addition, 24% of the sites did not reach 80% of their targeted sample size and this could be a major limit to generalize the results.

A total of 221 DBS and 17 plasma samples were analyzed. The amplification success rate was 55% for DBS (121/221) and 58.8% for plasma (10/17). The use of DBS remains common in resource-limited settings, though success rates can vary depending on sample handling, storage, and viral load [6,14,15,25]. The lower amplification rate observed could be attributed to nucleic acid degradation during storage/transport, or the presence of HIV-2. Indeed, two failed amplification specimens were serologically confirmed as HIV-2. Unfortunately, there was no possibility to reach the patients for a replacement sample due to the recruitment methodology used for this study.

The overall PDR rate was 10.7% (14/131), with NRTI resistance at 4.6% and NNRTI resistance at 9.2%. According to WHO, low PDR is <5%, intermediate 5–15%, and high >15%. The highest NNRTI PDR rates were observed in the Southeast (20.8%) and Dakar (13.6%), while the Southwest had 3.6%. These variations may reflect differences in sample size, patient care coverage, and local HIV prevalence (Southwest 1.5% vs Southeast 0.8%) [https://dhsprogram.com].

Importantly, the DRMs identified, particularly K103N among NNRTIs, are not expected to compromise the efficacy of dolutegravir-based regimens. However, they would have reduced the effectiveness of efavirenz-based ART, which was still widely used at the time of the survey [17]. This finding underscores both the limitations of NNRTI-based regimens and the rationale for the transition to dolutegravir (DTG) in Senegal [16].

The higher prevalence of NNRTI resistance observed in our study is consistent with previous reports from Senegal and other West African countries. This can be explained by the low genetic barrier of NNRTIs, their widespread use in first-line regimens until 2018, their administration for post-exposure prophylaxis in children, as well as their use in prevention of mother-to-child transmission programs. All of these factors would have contributed to the emergence and transmission of mutations such as K103N and Y181C [4,[6], [7], [8], [9],^26^].

The predominance of CRF02_AG (67.2%) aligns with previous reports in Senegal and West Africa [[19], [20]]. Unique recombinant forms and transmission chains were identified, highlighting the dynamic HIV epidemic in the country.

One limitation of this study is related to the lack of sequence data for the Protease gene as recommended by the WHO HIV drug resistance protocol (https://iris.who.int/bitstream/handle/10665/75204/WHO_HIV_2012.16_eng.pdf; accessed December 2023). Analyzing PR would have provided a more complete picture of HIVDR. Additionally, this study was conducted before the introduction of dolutegravir, so integrase sequencing was not performed, representing another limitation for assessing resistance to current first-line regimens. Finally, the proportion of HIVDR mutations was not considered, and subsequently, data could not be extrapolated to the entire country.

## Conclusion

Through a quasi-national survey, the level of PDR was estimated in Senegal and showed globally an intermediate level of resistant strains to NNRTI in PLHIV before initiating ART. These data revealed notable regional disparities, with the highest prevalence in the Southeast part of the country.

The prevalence of NRTI resistance, particularly to 3TC and Tenofovir Disoproxil Fumarate (TDF), suggests that the introduction of Tenofovir, Lamivudine, and Dolutegravir (TLD) in ART-naive patients could be considered with careful monitoring, rather than assuming guaranteed efficacy.

These findings also underscore the importance of ongoing surveillance and targeted attention in areas where the proportion of SDRM is not negligible, including for patients already on ART.

## Declaration of competing interest

The authors have no competing interests to declare.
